# Healthy Aging Index Report: Insights From 38 Major Chinese Cities

**DOI:** 10.1093/geroni/igaf122.354

**Published:** 2025-12-31

**Authors:** ShuangShuang Wang

**Affiliations:** Southwest Jiaotong University, Chengdu, Sichuan, China (People’s Republic)

## Abstract

The construction of a Health Aging Index is pivotal for evaluating cities’ readiness for an aging society. Utilizing the analytic hierarchy process, this study developed an index encompassing five areas (each score ranged 1-20): health care, living environment, transportation, social fairness and participation, and social security and finance, with 43 specific indicators. The evaluation of 38 Chinese large and medium size cities (bigger than 1 million population) revealed uneven development. Coastal cities, for instance, averaged 10.5 in health care, compared to 8.5 for some inland cities. Overall, Beijing led with a score of 58.5, while Guiyang scored 42.30, with a notably low score (2.47) in social security and finance. To foster healthy aging in China, tailored policies should be developed based on city-specific needs. Long-term care insurance systems must be strengthened to integrate medical and care services, and urban infrastructure should be enhanced to create age-friendly environments.

